# Impact of Prediabetes on In-Hospital Mortality and Clinical Outcomes in Acute Pancreatitis: Insights from a Nationwide Inpatient Sample

**DOI:** 10.3390/jcm14124271

**Published:** 2025-06-16

**Authors:** Tahani Dakkak, Nawras Silin, Riaz Mahmood, Shane S. Robinson, Nelson A. Royall

**Affiliations:** 1 Graduate Medical Education Research Department, Northeast Georgia Medical Center, Gainesville, GA 30501, USA; shane.robinson@nghs.com; 2 Department of Internal Medicine, Northeast Georgia Medical Center, Gainesville, GA 30501, USA; nawras.silin@outlook.com; 3 Department of Cardiology, Northeast Georgia Medical Center, Gainesville, GA 30501, USA; riaz.mahmood@nghs.com; 4 Hepato-Pancreato-Biliary Surgery, Department of Surgery, Northeast Georgia Medical Center, Gainesville, GA 30501, USA; nelson.royall@nghs.com

**Keywords:** acute pancreatitis, prediabetes, hospitalization outcomes, acute kidney injury, sepsis, national inpatient sample

## Abstract

**Background/Objectives**: Prediabetes is characterized by insulin resistance and systemic inflammation, which may increase susceptibility to acute pancreatitis (AP). However, limited data exist on how prediabetes influences in-hospital outcomes in AP patients. This study aimed to evaluate the prevalence and clinical outcomes of hospitalized AP patients with prediabetes using the National Inpatient Sample (NIS) database. **Methods**: We conducted a retrospective cohort study using NIS data from 2016 to 2018, identifying adult patients hospitalized with a primary diagnosis of AP. Patients were stratified based on the presence or absence of prediabetes; those with type 1 or 2 diabetes were excluded. The primary outcome is the association of prediabetes with developing acute pancreatitis and its influence on in-hospital mortality, length of stay, and total hospital cost. **Results**: Among 193,617 patients hospitalized with AP, 1639 had prediabetes. No statistically significant difference was found in in-hospital mortality, length of stay, or hospitalization costs between patients with or without prediabetes. The in-hospital mortality was 1.22% in prediabetic patients versus 2.01% in non-prediabetic patients (*p* = 0.0225). The length of stay was shorter in prediabetic patients (4.93 vs. 5.37 days, *p* = 0.0021), and hospitalization costs were similar (USD55,351.56 vs. USD57,106.83, *p* = 0.195). Furthermore, prediabetes was not an independent predictor of mortality (OR 0.50, 95% CI 0.31–0.82, *p* = 0.0063). Significant predictors of mortality included acute kidney injury (OR 12.98, 95% CI 11.96–14.09, *p* < 0.001) and severe sepsis with shock (OR 5.89, 95% CI 5.27–6.59, *p* < 0.001). **Conclusions**: Prediabetes was not associated with an increased in-hospital mortality in AP patients. However, complications such as AKI and septic shock significantly predicted mortality, underscoring the importance of early recognition and management.

## 1. Introduction

Acute pancreatitis (AP) is a common and potentially life-threatening gastrointestinal condition characterized by sudden inflammation of the pancreas. It accounts for 300,000 hospital admissions annually in the United States, making it one of the leading causes of gastrointestinal-related hospitalizations [1]. The clinical presentation of AP varies widely, ranging from mild, self-limiting disease to severe cases associated with multiorgan failure and significant mortality [2]. Several factors have been shown to influence disease severity, including advanced age, obesity, excessive alcohol intake, and the development of systemic inflammatory response syndrome (SIRS) [3].

Among the comorbidities associated with AP, diabetes mellitus has been extensively studied and linked to both an increased risk of developing the disease and poorer clinical outcomes [2,4]. Evidence suggests that individuals with type 2 diabetes mellitus have a threefold-increased risk of developing AP compared to those without diabetes [5]. This association is believed to be mediated by metabolic dysregulation, chronic low-grade inflammation, and increased susceptibility to pancreatic injury.

Despite the well-documented relationship between overt diabetes and AP, there remains a paucity of data regarding the role of prediabetes in this context. Prediabetes represents a transitional state between normoglycemia and type 2 diabetes, marked by insulin resistance and impaired glucose metabolism. In 2021, an estimated 97.6 million U.S. adults—over one-third of the adult population—were living with prediabetes [6]. This condition has been independently associated with systemic inflammation and organ dysfunction, raising concerns about its potential impact on diseases like AP. However, the current literature lacks robust data on the prevalence and outcomes of AP in patients with prediabetes.

Given the high and growing prevalence of prediabetes, understanding its influence on AP outcomes is of clinical importance. Identifying whether prediabetic patients are at increased risk for complications such as prolonged hospitalization, elevated healthcare costs, or in-hospital mortality may help guide triage decisions, risk stratification, and early interventions for high-risk groups [7].

The primary aim of this study is to evaluate the prevalence and clinical outcomes of hospitalized patients with AP and prediabetes using data from the National Inpatient Sample (NIS) database. Specifically, we sought to compare in-hospital mortality, length of stay (LOS), and total hospitalization costs between patients with and without prediabetes. Additionally, we aimed to identify independent predictors of in-hospital mortality among this patient population.

## 2. Materials and Methods

### 2.1. Data Source

This retrospective cohort study utilized data from the National Inpatient Sample (NIS), the largest publicly available all-payer inpatient healthcare database in the United States [8]. The NIS contains de-identified patient information, including clinical and non-clinical data, collected using International Classification of Diseases, Tenth Revision, Clinical Modification (ICD-10-CM) codes. Hospitalizations from the years 2016 to 2018 with a primary diagnosis of AP were identified using ICD-10-CM codes K85.0–K85.9. Patients were further classified into two cohorts: those with a secondary diagnosis of prediabetes (ICD-10-CM code R73.03) and those without this diagnosis. The diagnosis of prediabetes was based solely on ICD-10 coding without access to supporting laboratory data, such as HbA1c or fasting glucose, which limit diagnostic precision and may underrepresent the true prevalence of prediabetes in this population.

To isolate the effect of prediabetes, all patients diagnosed with type 1 or type 2 diabetes mellitus were excluded from the analysis using ICD-10-CM codes E10.1–E10.9 and E11.1–E11.9. Additional variables, including age, sex, race/ethnicity, and insurance type, were collected to characterize the study population. Clinical comorbidities analyzed included severe sepsis with shock, liver cirrhosis (K74.6), morbid obesity (E66.01), acute kidney injury (N17), stroke (I63), gastrointestinal bleeding (K92.2), acute coronary syndrome (I24), and atrial fibrillation (I48.0, I48.1, I48.2).

### 2.2. Study Outcomes

The primary outcomes were in-hospital mortality, length of hospital stays, and total hospitalization cost among patients admitted with AP, comparing those with and without prediabetes. A secondary objective was to identify independent predictors of in-hospital mortality in this patient population.

### 2.3. Statistical Analysis

Continuous variables were reported as means with standard deviations, and categorical variables as frequencies and percentages. Group comparisons for continuous variables (e.g., LOS, total cost) were conducted using independent *t*-tests. Associations between categorical variables (e.g., prediabetes status and in-hospital mortality) were analyzed using Chi-square tests. To evaluate independent predictors of in-hospital mortality, we performed multivariate logistic regression analysis. All statistical analyses were conducted using JMP Pro version 17 (SAS Institute Inc., Cary, NC, USA), with a significance level set at α = 0.001 to account for multiple comparisons.

### 2.4. Ethical Considerations

This study did not require institutional review board approval since it utilized publicly accessible de-identified data from the NIS database. All patient information was anonymized in compliance with the Health Insurance Portability and Accountability Act (HIPAA) guideline to ensure confidentiality.

## 3. Results

### 3.1. Patient Characteristics

Between 2016 and 2018, a total of 193,617 patients were diagnosed with AP, with a mean age of 51.76 ± 17.61 years. Among these, 1639 patients had prediabetes. The cohort of patients with prediabetes had a nearly equal distribution of males (50.81%) and females and was predominantly Caucasian (66.69%). Moreover, morbid obesity was significantly more common among prediabetic patients (11.95% vs. 4.57%, *p* < 0.001). Regarding insurance status, the most common types were private insurance (36.65%) and Medicare (34.33%). Additional demographic and clinical characteristics, including comorbidities, are summarized in Table 1.

### 3.2. In-Hospital Mortality

**Figure 1:**
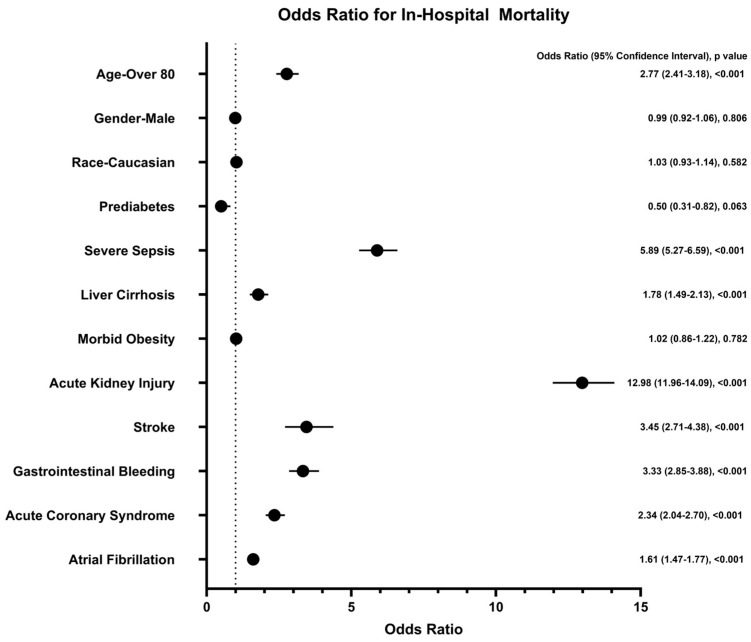
Odds ratio for in-hospital mortality. AKI and severe sepsis with shock were the strongest predictors of mortality in hospitalized patients with acute pancreatitis. On the other hand, prediabetes did not predict in-hospital mortality.

There was no statistically significant difference in overall in-hospital mortality between patients with and without prediabetes (1.22% vs. 2.01%, respectively; *p* = 0.0225), as presented in Table 2. However, certain comorbidities emerged as significant predictors of mortality in patients hospitalized with AP. Acute kidney injury (AKI) was the strongest predictor, with an odds ratio (OR) of 16.07 (95% confidence interval [CI]: 5.68–45.43; *p* < 0.0001), followed by severe sepsis with shock (OR 5.89, 95% CI: 5.27–6.59; *p* < 0.001). Other factors significantly associated with increased mortality included stroke (OR 3.45, 95% CI: 2.71–4.38; *p* < 0.0001), gastrointestinal bleeding (OR 3.33, 95% CI: 2.85–3.88; *p* < 0.0001), acute coronary syndrome (OR 2.34, 95% CI: 2.04–2.70; *p* < 0.0001), atrial fibrillation (OR 1.61, 95% CI: 1.47–1.77; *p* < 0.0001), and liver cirrhosis (OR 1.78, 95% CI: 1.49–2.13; *p* < 0.001). Advanced age, particularly in individuals over 80 years old, was also significantly associated with mortality (OR 2.77, 95% CI: 2.41–3.18; *p* < 0.001). Interestingly, morbid obesity was not associated with mortality in AP patients (OR 1.02, 95% CI: 0.86–1.22; *p* = 0.7824). These findings are illustrated in Figure 1.

### 3.3. Length of Stay and Total Hospitalization Costs

Patients with prediabetes had a slightly shorter mean LOS compared to those with prediabetes, averaging 5.37 days versus 4.93 days, respectively (*p* = 0.0021). However, this difference was not statistically significant. In terms of financial impact, the total hospitalization costs were similar among patients with and without prediabetes, with an average cost of USD57,106.83 compared to USD55,351.56 for those with prediabetes, indicating no statistically significant difference (*p* = 0.195). The differences in LOS and hospitalization costs are detailed in Table 2.

## 4. Discussion

This study aimed to evaluate the association between prediabetes and AP and its impact on LOS and mortality risk, using the National Inpatient Sample (NIS) database. Prediabetes could result from impaired fasting glucose or impaired glucose tolerance; however, we were unable to differentiate between subtypes of prediabetes due to reliance on the ICD-10 code available, which broadly defines prediabetes but does not distinguish the type. Our findings suggest that prediabetes did not negatively impact in-hospital mortality, LOS, or hospitalization costs compared to patients without prediabetes. This observation suggests that while prediabetes may have a less direct impact on mortality outcomes, it may still influence other aspects of hospital care. Interestingly, AKI, severe sepsis with shock, and other comorbidities emerged as significant predictors of mortality, highlighting critical factors that may play a larger role in determining patient outcomes.

While prediabetes is common among hospitalized patients with AP, the lack of association with mortality in our cohort could be attributed to multiple factors [9]. Prediabetes represents an intermediate stage of impaired glucose metabolism, which may not impact the clinical course of AP in the same manner as an established diagnosis of diabetes [10]. Previous research has suggested that individuals with prediabetes exhibit higher levels of C-reactive protein compared to those with normal glucose tolerance, indicating a heightened inflammatory response [5]. However, this inflammation is often manageable in prediabetic patients with preserved pancreatic function. While prediabetes may not be directly linked to mortality in AP patients, it is important to note that approximately 40% of individuals with prediabetes progress to diabetes over 5–10 years [9]. As prediabetes progresses to type 2 diabetes (T2D), the pancreas undergoes severe inflammatory damage characterized by increased release of pro-inflammatory cytokines, significantly compromising pancreatic function and increasing mortality in AP patients [11]. These findings underscore the importance of early detection and management of prediabetes, particularly in high-risk individuals, as timely intervention may mitigate progression to diabetes and reduce the risk of adverse outcomes in AP [9].

Despite the lack of association between prediabetes and mortality in our cohort, AKI and severe sepsis with shock emerged as strong predictors of mortality in patients with AP. The NIS dataset does not capture the timing of diagnosis or disease progression during hospitalization; therefore, it remains unclear whether these conditions contributed to the severity of AP or resulted from it. However, this finding aligns with existing literature, which recognizes AKI as a serious complication in up to 70% of patients with severe AP [12,13,14]. Several studies have reported that the development of AKI significantly worsens outcomes, with one study showing that 15% of patients with AKI required ICU admission [15,16]. Moreover, AKI has been found to double the mortality rate in patients with AP, emphasizing the critical nature of this condition [12]. The pathophysiology behind this association appears to be multifactorial. The release of pancreatic amylase from the injured pancreas may compromise kidney function by impairing renal microcirculation [17]. Additionally, complications such as hypoxia, hypovolemia, and abdominal compartment syndrome are believed to reduce renal perfusion pressure and worsen kidney function [17]. Therefore, early recognition and management of AKI in AP patients are essential for improving prognosis.

Severe sepsis with shock also appeared as a strong predictor of mortality in AP patients. Existing studies support this observation, showing that sepsis amplifies the inflammatory cascade, leading to impaired tissue perfusion and multi-organ dysfunction [18]. Prompt diagnosis and aggressive treatment of sepsis are crucial to prevent its progression to septic shock and subsequent multi-organ failure.

While findings such as the association of AKI and sepsis with increased mortality are well established in prior literature, their inclusion in the model served to validate the analysis and control for confounding influences. Furthermore, our study found that AP patients aged 80 and older were at significantly higher risk of mortality during hospitalization. This reflects the increased physiological vulnerability of older adults, which contributes to worse outcomes independent of AP severity. Previous research attributes this elevated risk to the pro-inflammatory state commonly observed in older adults, increased cytokine production in elderly patients with sepsis, and the frequent presence of multiple comorbidities that contribute to higher mortality risk [4,11,19,20,21]. This finding underscores the vulnerability of older patients to more severe outcomes in AP.

This study has several strengths. The use of a large, nationally representative dataset (NIS) and a stringent *p*-value threshold (*p* < 0.001) enhanced the robustness of our findings. Given the rare occurrence of AP, this extensive dataset was essential for providing a meaningful analysis of outcomes.

However, there are important limitations to consider. This study relied on ICD-10 coding, which may be subject to inaccuracies or misclassification. Additionally, the NIS database does not provide granular details on the severity of pancreatitis or specific management strategies used, both of which could influence outcomes. Medication history, including use of metformin or other diabetes therapies, was not available in the NIS dataset. Such treatments could potentially influence outcomes and represent a key area for future prospective studies. Lastly, due to its observational retrospective design, causal inferences cannot be made.

Future research should conduct propensity score matching to reduce confounding and examine the long-term effects of prediabetes on the progression and recurrence of AP, as well as explore whether early glycemic control interventions can improve clinical outcomes in this patient population.

## 5. Conclusions

To our knowledge, this is the first nationwide, inpatient-based study to investigate the association between prediabetes and AP in predicting mortality. Our study suggests that prediabetes does not have a significant impact on in-hospital mortality, LOS, or hospitalization costs in patients with AP. While prediabetes may not serve as a significant predictor of in-hospital mortality, clinicians should remain vigilant when managing patients with prediabetes due to the potential for long-term complications, including the risk of progressing to T2D. In contrast, established AKI, sepsis, and advanced age significantly influenced outcomes, underscoring the importance of these factors over prediabetes in the inpatient setting. Further research is needed to evaluate in-hospital factors that determine the course of AP in prediabetic patients. Understanding these relationships will help clinicians better manage and triage patients with AP, particularly those with metabolic conditions that may predispose them to poorer outcomes. Furthermore, future prospective studies are needed to characterize preexisting comorbidities before AP onset and examine their impact on clinical outcomes to allow for more accurate assessment of causality and disease progression.

## Figures and Tables

**Table 1:** Patient demographics and comorbidities of hospitalizations with acute pancreatitis with and without prediabetes.

Patient Characteristics	Prediabetes	No Prediabetes	*p*-Value
Age (years)			
Mean Years ± SD	56.53 ± 15.93	51.72 ± 17.61	<0.001
Gender			0.215
Male	807 (49.27%)	97,599 (50.82%)	
Female	831 (50.73%)	94,453 (49.18%)	
Race			<0.001
White	919 (57.58%)	123,873 (66.76%)	
Black	189 (11.84%)	27,424 (14.78%)	
Hispanic	330 (20.68%)	23,444 (12.64%)	
Asian	80 (5.01%)	3782 (2.04%)	
Native American	26 (1.63%)	1567 (0.84%)	
Other	52 (3.26%)	5449 (2.94%)	
Insurance			<0.001
Medicare	562 (34.33%)	57,763 (30.12%)	
Medicaid	318 (19.43%)	47,764 (24.91%)	
Private	600 (35.65%)	61,122 (31.87%)	
Self-Pay	111 (6.78%)	17,697 (9.23%)	
No Charge	6 (0.37%)	1653 (0.86%)	
Other	40 (2.44%)	5783 (3.02%)	
Comorbidities			
Stroke	6 (0.37%)	618 (0.32%)	0.753
Cardiogenic Shock	20 (1.22%)	2325 (1.21%)	0.973
Morbid Obesity	196 (11.95%)	8772 (4.57%)	<0.001
GI Bleed	9 (0.55%)	2017 (1.05%)	0.047
Acute Coronary Syndrome	21 (1.28%)	2134 (1.11%)	0.514
Atrial Fibrillation	102 (6.22%)	13,010 (6.77%)	0.375
Acute Kidney Injury	260 (15.85%)	28,363 (14.77%)	0.216
Liver Cirrhosis	18 (1.10%)	3476 (1.81%)	0.031
Severe Sepsis with Shock	20 (1.22%)	2325 (1.21%)	0.973

**Table 2:** Hospital admission outcomes in patients hospitalized with acute pancreatitis: comparison between those with and without prediabetes.

Outcome Measures	Prediabetes, N = 1639	Without Prediabetes, N = 191,978	*p*-Value
Length of Stay			
Mean number of days ± SD	4.93 ± 5.69	5.37 ± 7.60	0.0021
In-hospital Mortality			
Deceased	20 (1.22%)	3866 (2.01%)	
Survived	1619 (98.78%)	188,112 (97.99%)	0.0225
Total Hospital Costs			
Mean cost ± SD	USD 55,351.56 ± 81,371.87	USD 57,106,83 ± 132,918.38	0.195

## Data Availability

All data herein were derived directly from the National Inpatient Sample (NIS) database. The datasets used are available from the corresponding author upon request.

## Abbreviations

The following abbreviations are used in this manuscript:

AP	Acute Pancreatitis
NIS	National Inpatient Sample
ICD-10	International Classification of Diseases, Tenth Revision
AKI	Acute Kidney Injury
T2D	Type 2 Diabetes
ICU	Intensive Care Unit
LOS	Length of Stay
